# Supplementary material for: Interrogating Mutant Allele Expression via Customized Reference Genomes to Define Influential Cancer Mutations
Source: Sci Rep. 2019 Sep 4;9:12766. doi: 10.1038/s41598-019-48967-8 (PMC6726654; doi:10.1038/s41598-019-48967-8)
Supplement: Supplementary file 1 — Supplementary Figures 1-6 [file 41598_2019_48967_MOESM1_ESM.pdf]

Interrogating Mutant Allele Expression via Customized Reference Genomes to Define Influential Cancer Mutations

Adam D. Grant[1], Paris Vail[1], Megha Padi[2], Agnieszka K. Witkiewicz*[3], Erik S. Knudsen*[4]

1. University of Arizona Cancer Center, Tucson AZ 85719 USA
2. Department of Molecular and Cellular Biology, University of Arizona, Tucson, AZ, 85719 USA
3. Center for Precision Medicine, Roswell Park Cancer Center, Buffalo NY 14263 USA
4. Department of Molecular and Cellular Biology, Roswell Park Cancer Center, Buffalo NY 14263 USA

***Correspondence:**

Erik S. Knudsen

Department of Molecular and Cellular Biology

Roswell Park Cancer Center

Buffalo, NY 14263

erik.knudsen@roswellpark.org

Agnieszka K. Witkiewicz

Center for Precision Medicine

Roswell Park Cancer Center

Buffalo, NY 14263

agnieszka.witkiewicz@roswellpark.org

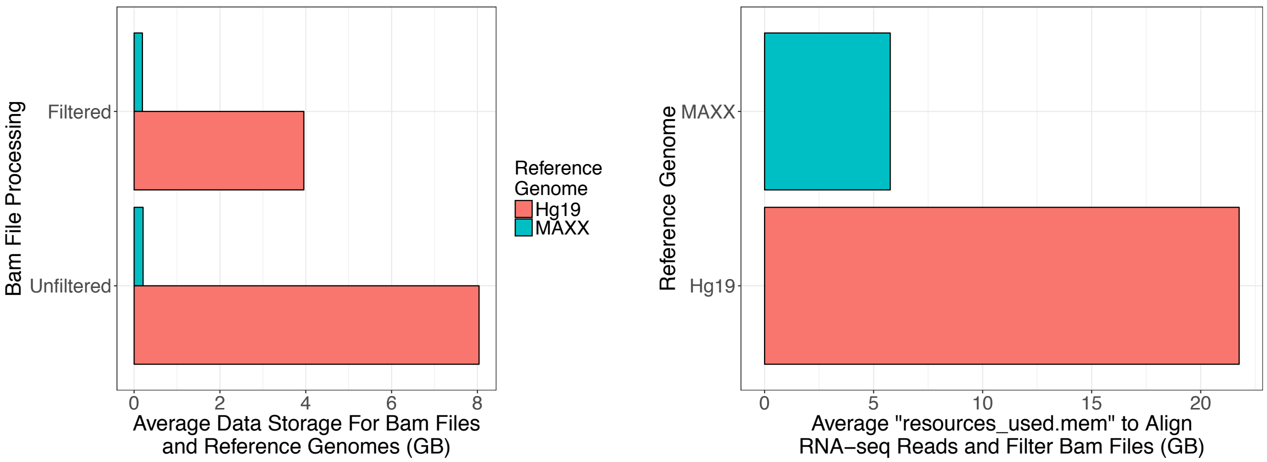

Supplementary Fig. 1: The average computational storage and processing used to perform allelic expression analysis using MAXX reference genomes and Hg19 reference genome. Filtered bam files underwent the removal of PCR duplicates, singleton reads, and reads that were not unique to specific loci.

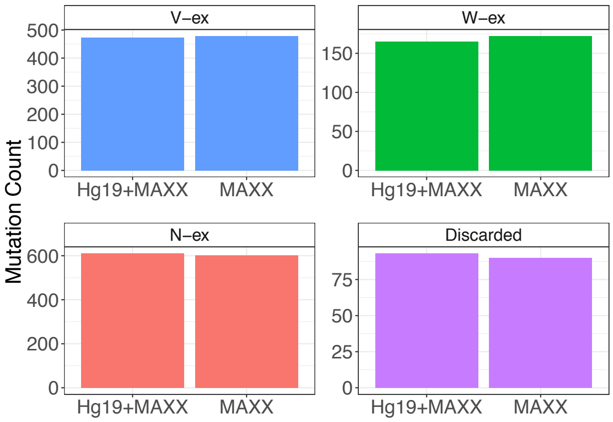

Supplementary Fig. 2: Comparison of mutation expression classification between the method of using a Hg19 + MAXX reference genome to using just a MAXX reference genome.

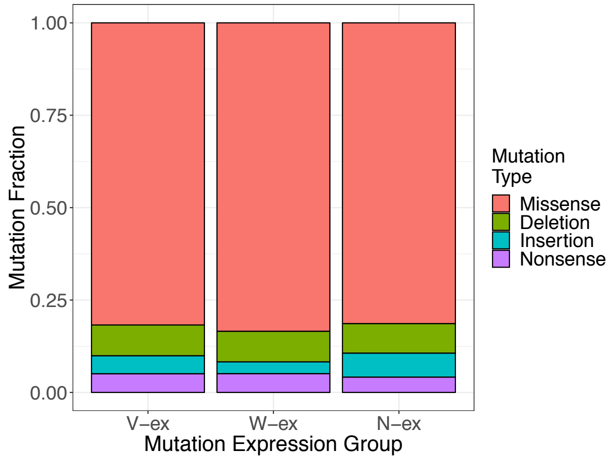

Supplementary Fig. 3: The fraction of different mutation types within each mutation expression group.

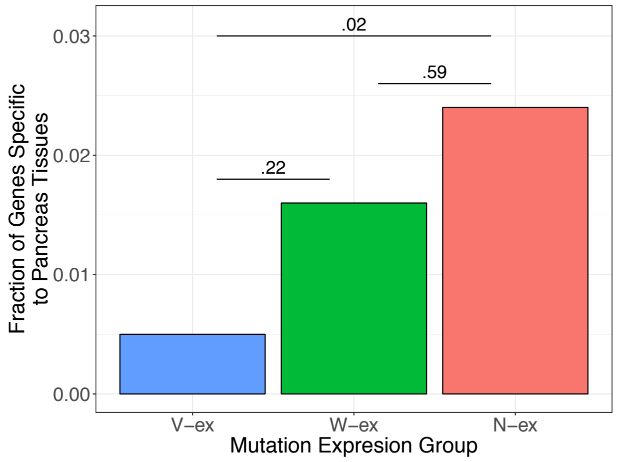

Supplementary Fig. 4: The fraction of pancreas tissue specific genes within each mutation expression group. A two-proportion z-test was used to determine statistical significance between each pairwise comparison of mutation expression groups.

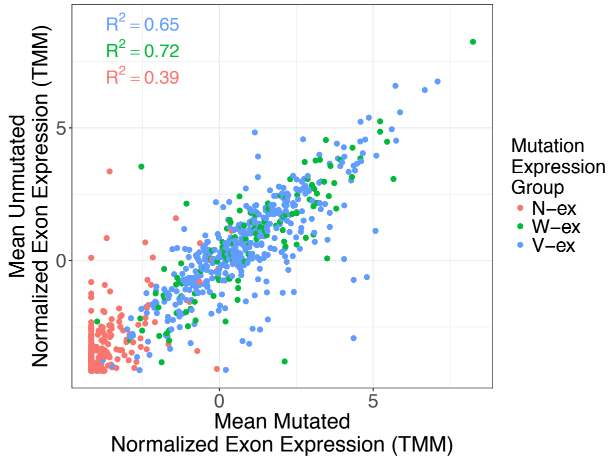

Supplementary Fig. 5: Comparison of the mean exon expression levels of samples that do contain the mutated exon to the mean exon expression levels of samples that don't contain the mutated exon.

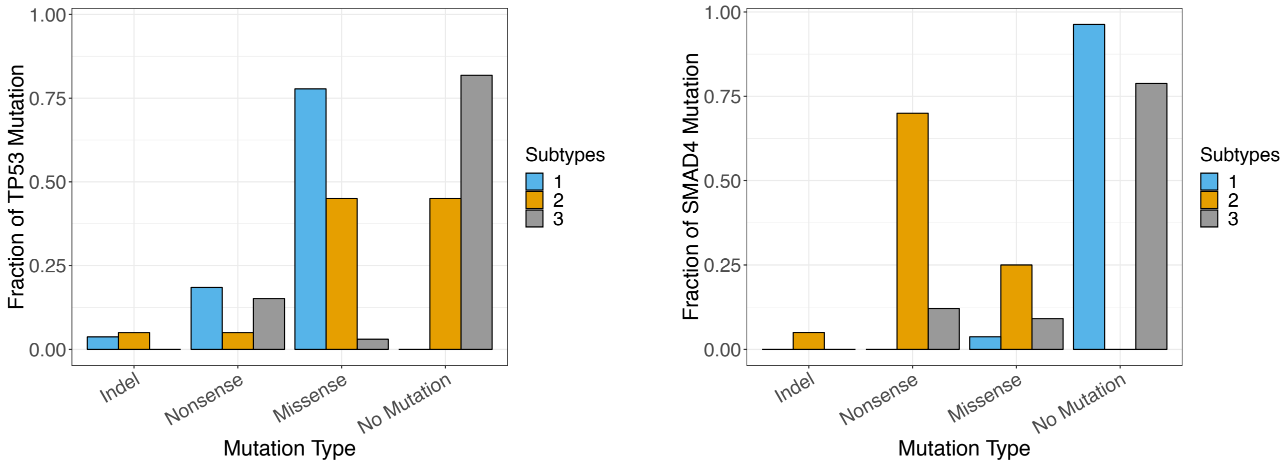

Supplementary Fig. 6: Fraction of PDAC patient derived cell lines and primary tumors classified as subtypes 1, 2, and 3 that have a TP53 or SMAD4 mutation.
